# Application value of overlap guiding tube (OGT) in assisting overlap esophagojejunostomy during laparoscopic total gastrectomy for gastric/gastroesophageal junction (G/GEJ) tumors

**DOI:** 10.1007/s10120-022-01296-8

**Published:** 2022-04-23

**Authors:** Chen Xinhua, Lin Tian, Huang Huilin, Zhao Mingli, Chen Tao, Chen Hao, Mai Jinsheng, Zhong Qinglei, Liu Hao, Zhao Liying, Hu Yanfeng, Li Guoxin, Yu Jiang

**Affiliations:** 1 Department of General Surgery and Guangdong Provincial Key Laboratory of Precision Medicine for Gastrointestinal Tumor, Nanfang Hospital, The First School of Clinical Medicine, Southern Medical University, Guangzhou, 510515 Guangdong China; 2 Clinical Medical School, Southern Medical University, Guangzhou, 510515 China

**Keywords:** Gastric/gastroesophageal junction (G/GEJ) tumors, Laparoscopy total gastrectomy, Overlap esophagojejunostomy, OGT

## Abstract

**Background:**

The overlap guiding tube (OGT) method, which was designed by our team to assist in overlap esophagojejunostomy, could potentially provide new perspectives for esophagojejunostomy. The application of the OGT-assisted method was first explored by our team and has not yet been reported.

**Methods:**

This cohort study analyzed the 3 month outcomes of 38 gastric/gastroesophageal junction (G/GEJ) tumor patients who underwent OGT-assisted overlap esophagojejunostomy.

**Results:**

There were 27 males and 11 females, aged 40–82 years. All patients underwent surgery successfully. The success rate of inserting anvil fork into esophageal lumen at first attempt was 97.4%. The total operation time, esophagojejunostomy time, volume of intraoperative blood loss, and length of surgical incision were 317.6 ± 51.5 min, 20.8 ± 3.8 min, 50.0 (range 15.0–200.0) ml, and 5.0 (range 4.0–8.0) cm, respectively. No procedures were converted to other laparoscopic anastomosis techniques or open approaches. The time to postoperative initial flatus, liquid diet intake, soft diet intake, and length of postoperative hospital stay were 3.0 (range 1.0–6.0) days, 4.0 (range 2.0–9.0)days, 6.0 (range 3.0–11.0) days, and 8.5 (range 6.0–16.0) days, respectively. Overall, postoperative complications were observed in 8 (21.1%) patients. Among them, one patients developed esophagojejunal anastomotic leakage. After 3 months of follow-up, none of the patients developed anastomotic stenosis or experienced unplanned secondary surgery or perioperative death.

**Conclusions:**

OGT-assisted overlap esophagojejunostomy for patients with G/GEJ tumors is safe and feasible, with good short-term effects. OGT method has a satisfactory success rate of inserting anvil fork into esophageal lumen at first attempt and could prevent from developing esophageal submucosa pseudocanals.

**Supplementary Information:**

The online version contains supplementary material available at 10.1007/s10120-022-01296-8.

## Background

In recent years, although the overall incidence of gastric cancer (GC) has declined, the incidence rate of proximal GC has increased [1, 2]. Surgical resection is the cornerstone of the treatment of advanced GC, and laparoscopic techniques are widely used due to their advantages, such as less invasiveness, rapid recovery, less bleeding and fewer complications [3–9]. At present, the feasibility of laparoscopic total gastrectomy (LTG) is still being explored, although the safety and effectiveness of laparoscopic radical gastrectomy for distal GC have been verified [3, 10]. The main reason is that performing esophagojejunostomy is the most challenging technical obstacle of LTG for surgeons to overcome [4, 11]. At present, there are many methods of esophagojejunostomy, but each has both its advantages and disadvantages, and there is no standard operation.

Conventionally, esophagojejunostomy is performed via mini-laparotomy at the upper epigastrium, which means difficult exposure and a narrow operation space, so an increasing number of surgeons have preferred total laparoscopic esophagojejunostomy in recent years. Among them, overlap esophagojejunostomy has gradually become one of the mainstream methods. Not only does overlap esophagojejunostomy not need purse-string sutures or insertion of an anvil, but also the size of the anastomosis stoma is also not limited by the transverse diameter of the esophagus and jejunum, which can largely prevent postoperative stenosis. Thus, overlap esophagojejunostomy has gradually become one of the mainstream methods. Since it was first reported in 2010 [12], the number of related studies about overlap esophagojejunostomy is increasing, and most have reported less severe anastomotic complications and satisfactory short-term outcomes [12–17].

However, the overlap operation is technically-difficult and time-consuming, and may result in some unique complications, such as the formation of esophageal submucosa pseudocanals. During the process, the controllability of inserting anvil fork into the esophageal mucosa canal through the hole in the esophagus is unstable. Therefore, we specifically designed an overlap guiding tube (OGT) to assist in increasing the stability of inserting anvil fork into the esophageal mucosa canal during the overlap esophagojejunostomy. The OGT connects the anvil fork with the nasogastric tube to form a connection device, thus stably and accurately guiding the insertion of the anvil fork into the esophageal mucosa canal through the esophageal hole with minimal size. Therefore, this study aims to assess the value of applying an OGT to assist with an overlap esophagojejunostomy during LTG in patients with gastric/gastroesophageal junction (G/GEJ) tumors.

## Methods

### Patients

From June to September 2021, 38 patients with G/GEJ tumors underwent with LTG with Roux-en-Y reconstruction, using the OGT-assisted overlap method of intracorporeal esophagojejunostomy, at Nanfang Hospital, Guangzhou, China. The inclusion criteria were as follows: (1) gastric cancer was confirmed by pathological examination; (2) had a tumor located in the GEJ or in the upper, upper to middle, or entire stomach; (3) had no obvious operative contraindication; (4) were aged 18–85 years; and (5) had an ECOG score of 0–2. Pathological staging was based on the TNM system of the 8th edition of the International Federation for the prevention and treatment of cancer [18]. This study meets the requirements of the Helsinki Declaration that was revised in 2013. Patients and their families signed informed consents before operation.

### Operative procedures

**Fig. 1:**
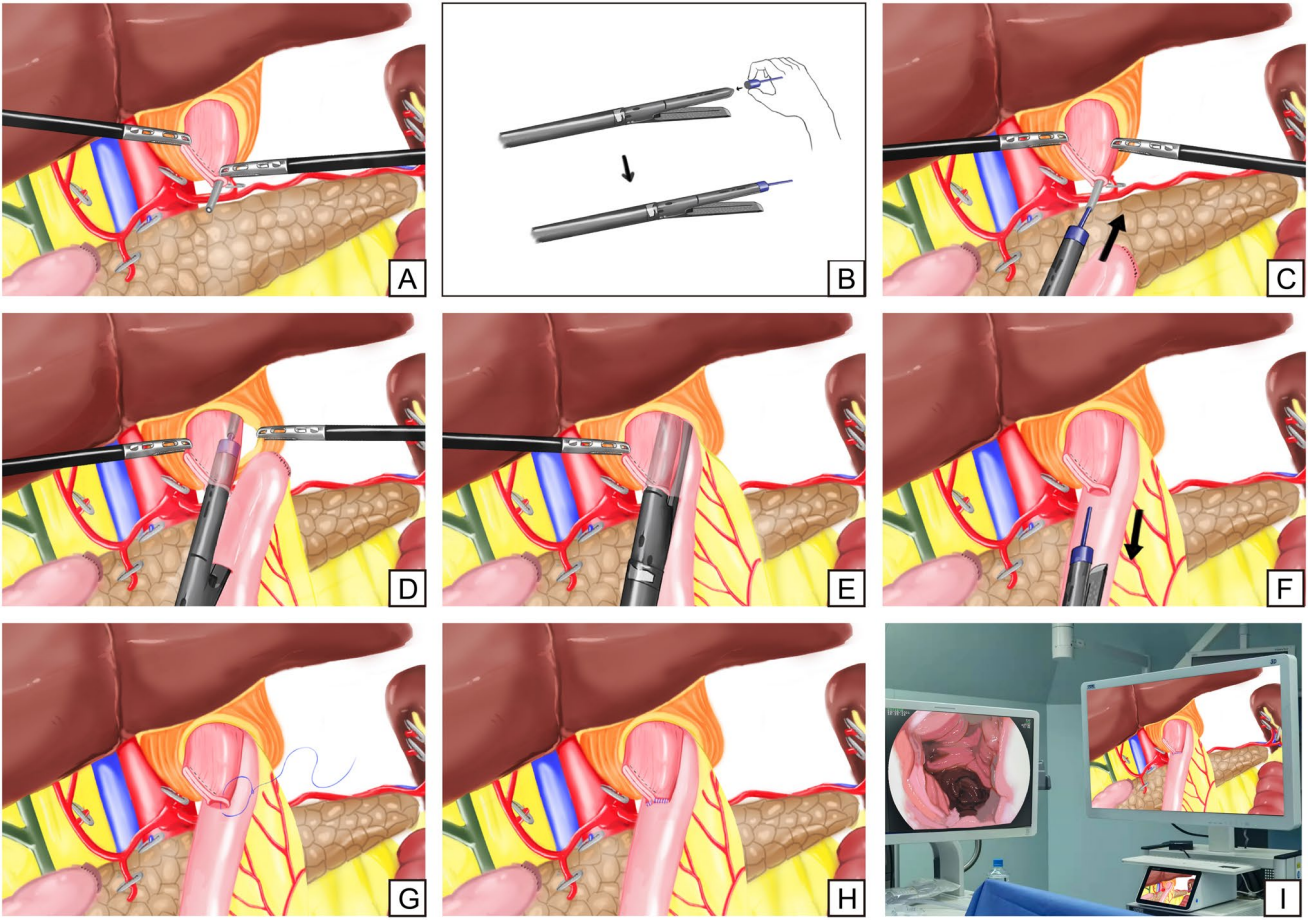
Outline of the OGT-assisted overlap method. **A** The nasogastric tube was pulled out 3 cm from the esophageal lumen to prepare to connect with OGT, **B** The OGT was sleeved on anvil fork extracorporeally, **C** Cartridge fork was inserted through jejunum opening toward the oral side of the lumen, while anvil fork sleeved with OGT was moved to connect with nasogastric tube, **D** While the stapler moved slowly toward the esophageal enterotomy by the surgeon, the anesthesiologist also adjusted the remaining length of the nasogastric tube synchronously, **E** By cooperation of surgeons and anesthesiologists, an integrated device formed by the connection of fork-OGT-nasogastric tube was moved carefully into the esophageal mucosa canal until the anvil fork was completely placed into the esophageal cavity, **F** After anastomosis, the OGT was withdrawn together with the anvil fork, **G** The common hole was made with minimized size, **H** The common hole was closed with full-thickness running suture using barbed sutures intracorporeally, **I** The anastomotic stoma was examined in both mucosa and serosa by endoscopy and laparoscopy. OGT: overlap guiding tube

**Fig. 2:**
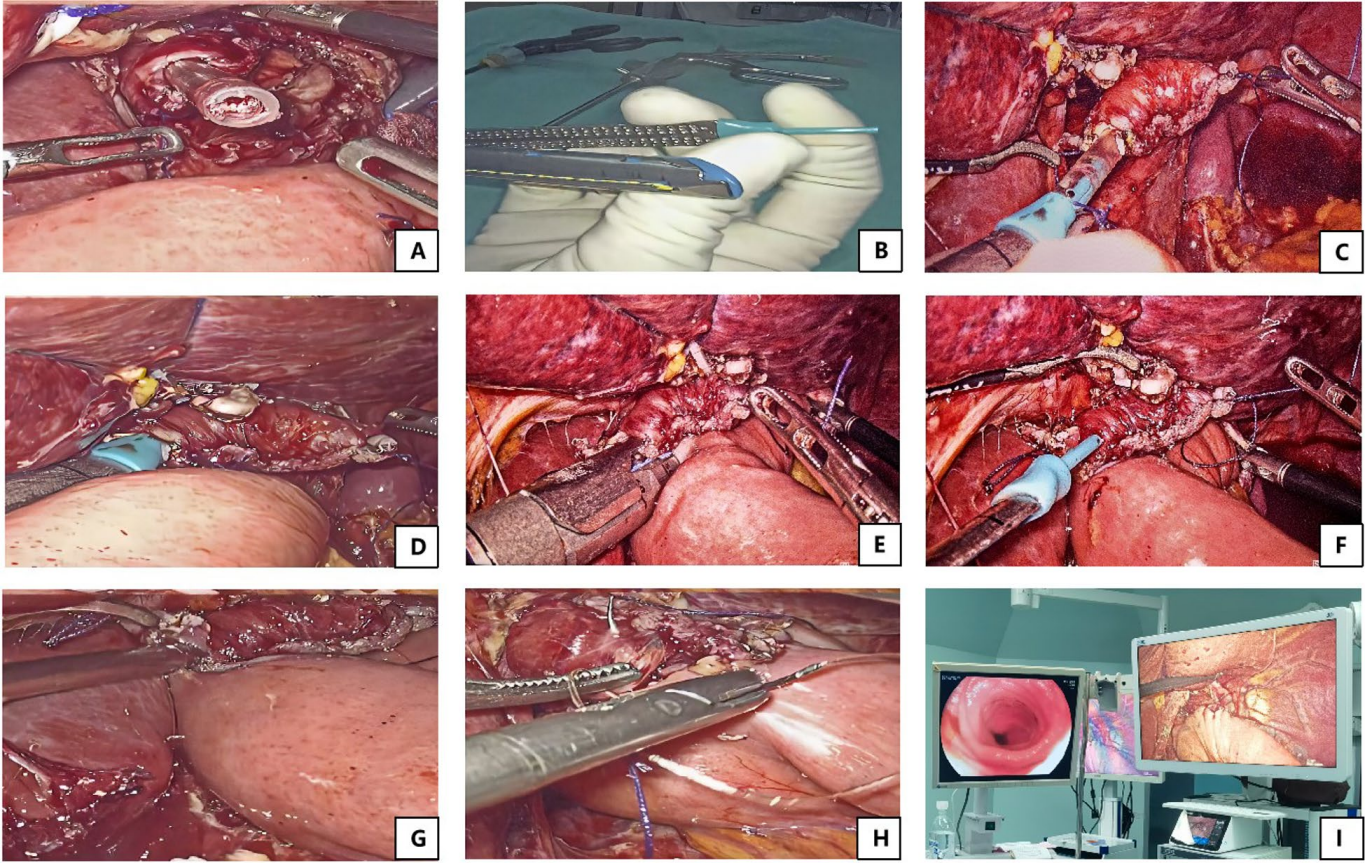
The process of the OGT-assisted overlap method. **A** The nasogastric tube was pulled out 3 cm from the esophageal lumen to prepare to connect with OGT, **B** The OGT was sleeved on anvil fork extracorporeally, **C** Cartridge fork was inserted through jejunum opening toward the oral side of the lumen, while anvil fork sleeved with OGT was moved to connect with nasogastric tube, **D**While the stapler moved slowly toward the esophageal enterotomy by the surgeon, the anesthesiologist also adjusted the remaining length of the nasogastric tube synchronously, **E **By cooperation of surgeons and anesthesiologists, an integrated device formed by the connection of fork-OGT-nasogastric tube was moved carefully into the esophageal mucosa canal until the anvil fork was completely placed into the esophageal cavity, **F** After anastomosis, the OGT was withdrawn together with the anvil fork, **G** The common hole was made with minimized size, **H **The common hole was closed with full-thickness running suture using barbed sutures intracorporeally, **I** The anastomotic stoma was examined in both mucosa and serosa by endoscopy and laparoscopy. OGT: overlap guiding tube

Under general anesthesia, gastrectomy was performed with D2 lymph node dissection by a laparoscopic approach [19, 20]. Then, the resected specimen was delivered via the extended umbilical incision, checked with free resection margins and immediately processed for further routine examination [21, 22]. Through the umbilical incision, the jejunum was transected at a point 20 cm distal to the ligament of Treitz using a linear stapler. At the lumen 50 to 55 cm distal from the site for planned esophagojejunostomy, a side-to-side jejunojejunostomy was performed using a linear stapler. The entry hole was closed with full-thickness running suture. Afterward, the pneumoperitoneum was reestablished to prepare to complete the subsequent intracorporeal esophagojejunostomy after closure of the umbilical incision using an incision protection device. A small enterotomy was made 5 cm distal to the stapler line on the antimesenteric side of the jejunal limb, while another small enterotomy was made on the posterior esophageal stump with the guidance of a nasogastric tube that was put into the esophageal lumen from the nose. After the enterotomy was made, the nasogastric tube was pulled out 3 cm from the esophageal lumen to connect with the OGT (Figs. 1A, 2A). Meanwhile, the OGT was sleeved on anvil fork extracorporeally (Figs. 1B, 2B).

After the linear stapler was put into abdomen through trocar, cartridge fork was inserted through jejunum opening toward the oral side of the lumen, while anvil fork sleeved with OGT was moved to connect with nasogastric tube (Figs. 1C, 2C). While the stapler moved slowly toward the esophageal enterotomy by the surgeon, the anesthesiologist also adjusted the remaining length of the nasogastric tube synchronously (Figs. 1D, 2D). By cooperation of surgeons and anesthesiologists, an integrated device which was formed by the connection of the fork-OGT-nasogastric tube, was moved carefully into the esophageal mucosa canal until the anvil fork was completely placed into the esophageal cavity and adjusted to a satisfactory angle to perform side-to-side esophagojejunostomy (Figs. 1E, 2E). Then, the anesthesiologist continued to pull back the nasogastric tube for 10 cm to ensure that the stapler would not clamp the nasogastric tube. After that, the surgeon began to fire the stapler to perform side-to-side esophagojejunostomy. After anastomosis, the OGT was withdrawn together with the anvil fork (Figs. 1F, 2F). Then the common hole was made with minimized size (Figs. 1G, 2G). Finally, the common hole was closed with full-thickness running sutures using barbed sutures intracorporeally (Figs. 1H, 2H). After esophagojejunostomy, the anastomotic stoma was examined in both mucosa and serosa by endoscopy and laparoscopy (Figs. 1I, 2I). The esophagojejunostomy process is shown in Video 1.

### Data collection and outcome assessment

Demographic characteristics were collected from all the patients, including age, sex, body mass index (BMI), comorbidities, physical status according to the Eastern Cooperative Oncology Group (ECOG), pathological features, and preoperative laboratory values comprising hemoglobin, total proteins, and albumin. Tumor staging was reported according to the 8th edition of the International Union Against Cancer (UICC) TNM Classification [18].

Surgery-related indices included the surgery time, time of digestive tract reconstruction, time of esophagojejunostomy, success rate of inserting anvil fork into esophageal lumen at first attempt, number of attempts to insert anvil fork into esophagus, estimated blood loss, number of retrieved LNs, tumor size, proximal margin of the tumor, incision length and intraoperative complications. Postoperative conditions included overall complications and anastomotic complications, early postoperative recovery, length of postoperative hospital stay, perioperative unplanned secondary operation rate and perioperative death.

Follow up: the patients were followed up by outpatient visits and telephone interviews. The follow-up time lasted until December 2021. Postoperative complications were defined as problems affecting patients during their hospital stay following surgery, rated in accordance with the Clavien–Dindo classification (CDC) system [23].

The definitions of some indices are as follows. (1) Esophagojejunostomy time: time from making the entry hole for the anastomosis on the esophageal stump to the time the common entry hole was closed and reinforced using barbed threads. (2) Inserting anvil fork into esophageal lumen at first attempt: when inserting the anvil fork into the esophageal hole, the anvil fork can be correctly placed in a satisfactory position and at a satisfactory angle into the esophageal mucosa canal to be fired for esophagojejunostomy by inserting it only once. (3) The number of attempts to insert anvil fork into esophagus: the number of times required to try to insert the anvil fork in a satisfactory position and at a satisfactory angle into the esophageal mucosa canal to be fired for esophagojejunostomy.

### Statistical analysis

The data were analyzed using the SPSS version 25.0 (SPSS Inc., Chicago, IL, USA). Continuous variables with normal distribution are expressed as the mean ± standard deviation, continuous variables with a skewed distribution are expressed as the median (range). Shapiro–Wilk test were performed to confirme the normal/skewed distribution of the continuous variables. While categorical variables are expressed as the number and percentage.

## Results

### Demographic and clinical characteristics

As shown in Table 1, there were 27 males and 11 females; 38 patients were aged from 40–82 years, with a median age of 60.5 years. The BMI was 22.6 ± 3.2 kg/m^2^. Among them, 4 patients had a history of abdominal surgery, 5 patients had tumor-related bleeding, 4 patients had tumor-related obstruction, 12 patients received neoadjuvant therapy, and 21 patients had tumors invading the esophagus. The most common comorbidities were respiration diseases (12 of 38, 31.60%). All patients had an ECOG ≤ 2. The serum albumin, total protein, and hemoglobin levels of the 38 patients were 37.4 ± 3.8 g/L, 62.9 ± 6.3 g/L and 116.6 ± 23.0 g/L, respectively.

**Table 1:** Demographic and clinical characteristics of the patients

	*N* = 38
Age (years, mean ± SD)	60.5 ± 9.5
Sex(M/F)	27/11
Body mass index (kg/m^2^, mean ± SD)	22.6 ± 3.2
ECOG [*n* (%)]	
0	15(39.5)
1	10(26.3)
2	13(34.2)
Previous abdominal surgery [*n* (%)]	4(10.5)
Comorbidity [*n* (%)]	
Diabetes	5(13.2)
Hypertension	7(18.4)
Respiratory	12(31.6)
Cardiovascular	7(18.4)
Tumor complications [*n* (%)]	
Bleeding	5(13.2)
Obstruction	4(10.5)
Serum albumin (g/L, mean ± SD)	37.4 ± 3.8
Total protein (g/L, mean ± SD)	62.9 ± 6.3
Hemoglobin (g/L, mean ± SD)	116.6 ± 23.0
Neoadjuvant therapy [*n* (%)]	12(31.6)
Invaded esophagus [*n* (%)]	21(55.3)
cT category [*n* (%)]	
T1	4(10.5)
T2	9(23.7)
T3	11(28.9)
T4	14(36.8)
cT category [*n* (%)]	
N0	13(34.2)
N1	10(26.3)
N2	7(18.4)
N3	8(21.1)
cM category [*n* (%)]	
M0	37(97.4)
M1	1(2.6)

### Operation situation

As shown in Table 2, all 38 patients underwent surgery successfully. Among them, 36 patients underwent R0 resection. 36 patients underwent D2 lymphadenectomy and 2 patients underwent D2 + lymphadenectomy. All patients achieved a R0 proximal resection margin, and the proximal cutting edge was 2.0 (range 0.1–10.0) cm. The success rate of inserting anvil fork into esophageal lumen at first attempt was 97.4% (37 of 38). The patient in whom the device failed to be placed in the esophagus expectedly at first attempt because the cartridge fork punctured the jejunum after we placed OGT device into the esophagus at first attempt. Thus, we pull out the OGT and anvil fork from esophagus, and cartridge fork from the jejunum. Then, we sutured the puncture the jejunum and then put fork and OGT into esophageal lumen with success at second attempt. Attempts of inserting anvil fork into esophagus was 1 (range 1–2). No procedure was converted to other laparoscopic anastomosis techniques or open approaches. The total operation time, esophagojejunostomy time, number of retrieved lymph nodes, volume of intraoperative blood loss, and length of surgical incision of 38 patients were 317.6 ± 51.5 min, 20.8 ± 3.8 min, 46.0±14.3, 50.0(range 15.0–200.0) ml, and 5.0(range 4.0–8.0) cm, respectively. Intraoperatively, one patient experienced jejunum puncture, and another patient confronted pleural tear (Table 2). However, encouragingly, none of them encountered intraoperative anastomotic troubles.

**Table 2:** Surgical features and pathological characteristics

	*N* = 38
D2/D2 + lymphdenectomy	36/2
Combined resection [*n *(%)]	1
R0/R1-2 resection [*n* (%)]	36/2
Tumor size (cm, mean ± SD)	3.6 ± 1.9
No. of retrieved lymph nodes (mean ± SD)	46.0 ± 14.3
(y) pT category [*n* (%)]	
ypT0	6(15.8)
(y)pT1	9(23.7)
(y)pT2	8(21.1)
(y)pT3	12(31.6)
(y)pT4	3(7.9)
(y) pN category [*n* (%)]	
(y)pN0	27(71.1)
(y)pN1	1(2.6)
(y)pN2	5(13.2)
(y)pN3a	4(10.5)
(y)pN3b	1(2.6)
(y) pM category [*n* (%)]	
(y)pM0	37(97.4)
(y)p M1	1(2.6)
R1/R2 proximal resection margin [*n* (%)]	0
Proximal margin (cm, M (range))	2.0(0.1–10.0)
Conversion to open surgery [*n* (%)]	0
Conversion to other laparoscopic anastomosis techniques [*n* (%)]	0
Total operative time (min, mean ± SD)	317.6 ± 51.5
Esophagojejunostomy time† (min, mean ± SD)	20.8 ± 3.8
Insert anvil fork into esophageal lumen at first attempt [*n* (%)]	37(97.4)
Attempts of inserting fork into esophagus (M(IQR))	1(1–2)
Intraoperative complications [*n* (%)]	2(5.3)
Jejunum was punctured	1(2.6)
Pleural was torn	1(2.6)
Intraoperative anastomotic trouble	0
Blood loss (mL, M(range))	50.0(15.0–200.0)
Incision length (cm, M(range))	5.0(4.0–8.0)

^†^OGT-assisted Overlap esophagojejunostomy time: defined as time from making the entry hole for the anastomosis on the esophageal stump to the common entry hole was closed

### Postoperative conditions

As shown in Table 3, the time of first ambulation, time to postoperative initial flatus, time to postoperative initial liquid diet intake, time to postoperative initial soft diet intake, time to pull drainage, and postoperative hospital stay of 38 patients were 1.0 (range 1.0–3.0) days, 3.0 (range 1.0–6.0) days, 4.0 (range 2.0–9.0) days, 6.0 (range 3.0–11.0) days, 6.0 (range 4.0–14.0) days, and 8.5 (range 6.0–16.0) days, respectively. Overall, postoperative complications were observed in 8 (21.1%) patients (Table 4). Among them, one patient developed esophagojejunal anastomotic leakage (EJAL), which was managed by endoscopic treatment with a self-expanding metal stent. However, the 3 month follow-up did not reveal any anastomotic stenosis. No patient developed esophagojejunal anastomotic bleeding, but one patient suffered from jejunal-jejunal anastomotic stoma bleeding. Three patients (7.9%) developed abdominal infection and were cured by drainage and anti-infection medication. Mild pneumonia was the most common (5 of 38, 13.2%) postoperative complication and could be cured by conservative treatment such as anti-infection and promotion of sputum excretion. All 38 patients were followed up for 3 months. None of them developed anastomotic stenosis or required unplanned secondary surgery or experienced perioperative death.

**Table 3:** Short-term surgical outcomes after laparoscopic total gastrectomy with esophagojejunostomy constructed by OGT-assisted Overlap

	*N* = 38
Time of first ambulation (d, M (range))	1.0 (1.0–3.0)
Time to first flatus (d, M (range))	3.0 (1.0–6.0)
Time to liquid diet (d, M (range))	4.0 (2.0–9.0)
Time to soft diet (d, M (range))	6.0 (3.0–11.0)
Time to pull drainage (d, M (range))	6.0 (4.0–14.0)
Length of postoperative hospital stays (d, M (range))	8.5 (6.0–16.0)

**Table 4:** Postoperative complications

	*N* = 38
Postoperative complications [*n* (%)]	8(21.1)
EJ-related complications	1(2.6)
Anastomotic leakage	1(2.6)
Anastomotic stenosis	0
Anastomotic bleeding	0
J‐J complications	1(2.6)
J‐J leakage	0
J-J stenosis	0
J-J bleeding	1(2.6)
Respiraion infection	5(13.2)
Abdominal infection	3(7.9)
Clavien–Dindo classification [n (%)]	
I	0
II	7(18.4)
IIIa	1(2.6)
Unplanned secondary surgery [n (%)]	0
Perioperative death [n (%)]	0

## Discussion

The OGT-assisted method was designed for overlap esophagojejunostomy to avoid repeated insertions of anvil fork into esophageal lumen, and to prevent  the development of esophageal submucosa pseudocanals. The formation of an integrated device connected by OGT theoretically could increase success rate of inserting anvil fork into esophageal lumen at first attempt, thus reducing the risk of esophageal injury. Additionally, it could completely prevent from developing a “false canal”. Furthermore, the connection and synchronous movement of the anvil fork and nasogastric tube could prevent the anvil fork from stabbing the esophageal wall or cutting the nasogastric tube when the stapler is fired within the lumen. These technical characteristics of OGT significantly improve the controllability and safety of inserting the anvil fork into the esophagus. Therefore, when cutting the hole of the esophagus to insert the anvil fork, we could minimize the size of the hole, thus reducing the difficulty of closing common hole and then shortening the time of suturing the entry hole by barbed threads, and then reducing the risk of anastomotic defects. However, OGT-assisted method was first explored by our team and has not yet been reported. Thus, we conducted this study to explore the application value of OGT in assisting overlap esophagojejunostomy in LTG for G/GEJ tumors.

In all 38 patients, the success rate of inserting anvil fork into esophageal lumen at first attempt was 97.4%. The discrepancey about the success rate of inserting anvil fork into esophageal lumen at first attempt between OGT-overlap method and traditional overlap is apprant in surgeons` experiences. However, the previous reports did not notice this variable since repeatedly inserting of anvil fork into esophageal lumen are default situation in clinical practices. Obviously, the increase of success rate of inserting anvil fork into esophageal lumen at first attempt by OGT greatly reduced the risk of esophageal injury. Of course, in next step, we should focus on the comparisions about success rate of inserting anvil fork into esophageal lumen at first attempt between OGT-overlap method and traditional overlap method in the comparative studies and randonmised clinical trials.

As expected, none of the patients developed esophageal submucosal pseudocanals. These advantages were achieved by the operation of moving the connection of the fork-OGT-nasogastric tube into the esophageal mucosa. The mean time of OGT-assisted overlap esophagojejunostomy was 20.8 ± 3.8 min. It is much shorter than that in most previous studies, which reported that the esophagojejunostomy time of traditional overlap esophagojejunostomy ranged from 34.3 to 45 min [17, 24–26]. These data indicated that the OGT-assisted overlap procedure is simplified, stable and easily mastered. The main factors that affected the time of overlap esophagojejunostomy included the operational proficiency and the cooperation of the surgeons’ team, the length of esophageal dissociation, BMI, and the suspension of liver, which cannot be compared homogeneously across different studies. However, in clinical practice, we found that the shortening of OGT-assisted overlap esophagojejunostomy time mainly involves two other unique aspects: (1) It is easier and more stable to insert the anvil fork into the esophageal cavity by docking the anvil fork with the nasogastric tube via the OGT. In this study, when the anvil fork was inserted into the esophageal hole, the guidance of OGT not only ensured that the anvil fork could be completely inserted into esophageal mucosa canal, but also helped the anvil fork to be correctly inserted at a satisfactory position and a satisfactory angle into the esophageal cavity to immediately fire for esophagojejunostomy; the success rate of inserting anvil fork into esophageal lumen at first attempt was 97.4%, so the insertion times and time of inserting anvil fork into the esophageal lumen can be shortened and be more stable; (2) Because the OGT worked in achieving satisfactory uccess rate of inserting anvil fork into esophageal lumen at first attempt and prevents from developing esophageal submucosa pseudocanals, thus reducing esophageal injury and operative complications, the surgeons reserved the time to address these issues; (3) Since OGT can ensure that the anvil fork is placed in the true esophageal mucosa canal without worrying about the formation of an esophageal submucosa pseudocanal, surgeons could minimize the size of common hole and thus shorten the time to suture the common hole.

Anastomotic safety is a major concern for surgeons after total gastrectomy. Insecure anastomosis may cause severe complications, especially EJAL, which will prolong the postoperative hospital stay, increase medical costs, aggravate the risk of anastomotic stricture, increase the need for reoperation, cause morbidity-related death, and even affect long-term prognosis [26–30]. According to the nationwide internet-based database of Japan, the incidence of anastomotic leakage after total gastrectomy was 4.4% (881 of 20,011) in 2011[31]. Another study from 1997 to 2016 showed the incidence of EJAL was 6% (58 of 969) [30]. Schietroma et al. reported an incidence of EJAL up to 14.6% [32]. For patients with Siewert type 2 adenocarcinoma of the esophagogastric junction, the incidence of EJAL in our center reached 13.5% [4]. Therefore, in the clinical setting, EJAL is regarded as one of the most critical postoperative complications. Thus, we consider it an important evaluation index for the safety of overlap esophagojejunostomy [33, 34]. Whether OGT sleeved in anvil fork will affect the quality and safety of anastomotic stoma is a concern for surgeons. We have previously confirmed in the animal experiments that the OGT sleeved in anvil fork does not affect the firing of stapler, nor does it affect the formation of anastomotic stoma. In this study, only one patient (1 of 38, 2.6%) developed EJAL, which is a much lower incidence than the previous report of the incidence of EJAL after gastrectomy. Furthermore, there was no anastomotic bleeding or anastomotic stenosis after 3 months of follow-up. Furthermore, we notice that many studies have confirmed that invading esophagus and receiving neoadjuvant therapy are two main risk factors of EJAL [11, 35]. In our study, 12 patients (31.6%) received neoadjuvant therapy, and in 21 patients (55.3%), the esophagus was invaded. Therefore, we concluded that OGT-assisted overlap esophagojejunostomy does not affect the quality of anastomosis, and it is a safe and feasible optimization method, even for patients who are at risk of EJAL.

The limitations of this method are the potential risk that OGT may drop into the abdomen during surgery, and that stapler may cut into nasogastric tubes. To solve the worrying about the potential risk of OGT dropping into the abdomen during surgery, we have taken several measures: (1) We have adjusted the size of the OGT for many times to best fit the size of anvil fork in the product development process. At present, the size of OGT allows the OGT to tightly connect with anvil fork, and minimizes the risk of the spontaneous dropping of OGT during the anastomosis process. (2) Before sleeving the anvil fork with OGT, we don’t lubricate the anvil fork with paraffin oil, which will cause the OGT to slip; (3) To avoid the neglecting of the situation that OGT drops into the abdomen, we instructed nurses to confirm whether the OGT is still sleeved in the anvil fork and the OGT must be retrieved once the stapler is pulled out. (4) To further prevent the OGT from dropping in and becoming lost in the abdominal cavity, we also changed the color of OGT from white at the beginning to its current more conspicuous green color. If the OGT drops into the abdominal cavity, the conspicuous green color will allow it to be found quickly. With these efforts, none of the patients in this study experienced a situation in which the OGT was lost within the abdominal cavity.

In addition, to solve the worrying about the potential risk that stapler may cut into nasogastric tubes when the stapler is fired for esophagojejunostomy, several measures were taken: (1) In terms of the operation process, we set rules that after the OGT connects with the nasogastric tube, we required that the anesthesiologists retreat the nasogastric tube at the same time as when the surgeons move the stapler slowly toward the esophageal hole. Because of the surgeons` and anesthesiologists` cooperation, the integrated device formed by the fork-OGT-nasogastric tube connection is moved into the esophageal mucosa canal, and the nasogastric tube is prevented from being folded in the anastomotic area and clamped by the stapler. (2) To further confirm that the nasogastric tube is not clamped by the stapler, before firing the stapler, the surgeons must  require the anesthesiologists to retreat the nasogastric tube by 10 cm (to ensure that it exceeds the length of the stapler fork), and then fire the linear stapler to implement side-to-side esophagojejunostomy; (3) Moreover, we are developing a harder nasogastric guiding catheter to replace the current nasogastric tube that connects with OGT. This change will not only help surgeons cut the esophageal hole faster and more accurately, but will also prevent stapler clamping of the nasogastric tube since the stapler can not clip and fire when clamping the hard nasogastric guiding catheter. A harder nasogastric guiding catheter will make the OGT-assisted method simpler and safer.

## Conclusions

Therefore, OGT-assisted overlap esophagojejunostomy for patients with G/GEJ tumors is safe and feasible, with good short-term effects. The OGT-assisted method provides new perspectives for esophagojejunostomy.

## Supplementary Information

Below is the link to the electronic supplementary material.

Supplementary file1 (MP4 102513 KB)

## Data Availability

The raw data supporting the conclusions of this article will be made available by the authors upon reasonable request. Requests to access the datasets should be directed to balbc@163.com.
